# Pathological alterations in striatal compartments in the human brain of autism spectrum disorder

**DOI:** 10.1186/s13041-020-00624-2

**Published:** 2020-05-27

**Authors:** Hsiao-Ying Kuo, Fu-Chin Liu

**Affiliations:** Institute of Neuroscience, National Yang-Ming University, 155, Sec. 2, Li-Nong Street, Taipei, 11221 Taiwan

**Keywords:** Basal ganglia, Striatum, Caudate nucleus, Striosome, Autism

## Abstract

The striatum comprises a mosaic structure of striosomal and matrix compartments. Imbalanced neuronal activity between striosomes and matrix is implicated in neurological deficits in psychomotor and limbic functions. Because patients with autism spectrum disorder (ASD) are impaired in social communication and psychomotor function, it raises the possibility that abnormal striatal compartments may contribute to ASD pathogenesis. Here, we provide pathological evidence from human postmortem brains to support this hypothesis. Because ASD is a neurodevelopmental disease that emerges early in childhood, we analyzed juvenile and adolescent brains. Distinct patterns of *PRODYNORPHIN*-positive and calbindin-poor striosomes were detected in the caudate nucleus of control brains by in situ hybridization and immunohistochemistry. By contrast, *PRODYNORPHIN*-positive and calbindin-poor striosomes were decreased in the caudate nucleus of young ASD brains. Moreover, calbindin, a matrix marker, was aberrantly increased in the striosomal compartment, obscuring the boundaries between calbindin-poor striosomes and calbindin-rich matrix in ASD caudate nucleus. Calbindin-positive cells were decreased in the ASD matrix compartment. Collectively, our study has uncovered for the first time that aberrant striatal compartments occur in the caudate nucleus of human ASD brains, which suggests abnormal striatal compartmentation as a pathological signature that has previously been underestimated in ASD pathogenesis.

## Main text

Autism spectrum disorder (ASD) is characterized by deficits in social communication and repetitive behavior, and the etiology of ASD is complex and heterogeneous [1]. The basal ganglia circuits are engaged in motor control, including action selection and reinforcement learning [2]. The caudate nucleus and putamen of dorsal striatum in basal ganglia comprise two neurochemical compartments, striosome/patch and the matrix [3]. Clinical studies have linked dysfunction of basal ganglia to ASD pathogenesis [4]. The faster growth rate of the caudate nucleus is correlated with the severity of repetitive behaviors of ASD patients [5] and the activity of the caudate nucleus of ASD patients is altered in response to reward [6]. Interestingly, animal studies have shown that neuronal activity of striosomes is involved in reinforcement and reward learning [7–9]. Aberrant striatal compartments have been observed in an ASD mouse model [10]. These findings raise an intriguing possibility that dysfunctional striosomal circuits may be involved in the pathophysiology of ASD.

In the present study, we investigated whether striatal compartmentation is altered in human ASD brains. We analyzed juvenile and adolescent brains, because ASD is a neurodevelopmental disease and ASD symptoms emerge early in childhood [1]. We focused on the caudate nucleus, as the caudate nucleus is involved in cognitive and motor functions and striosomal organization is prominent in this structure [11].

**Fig. 1:**
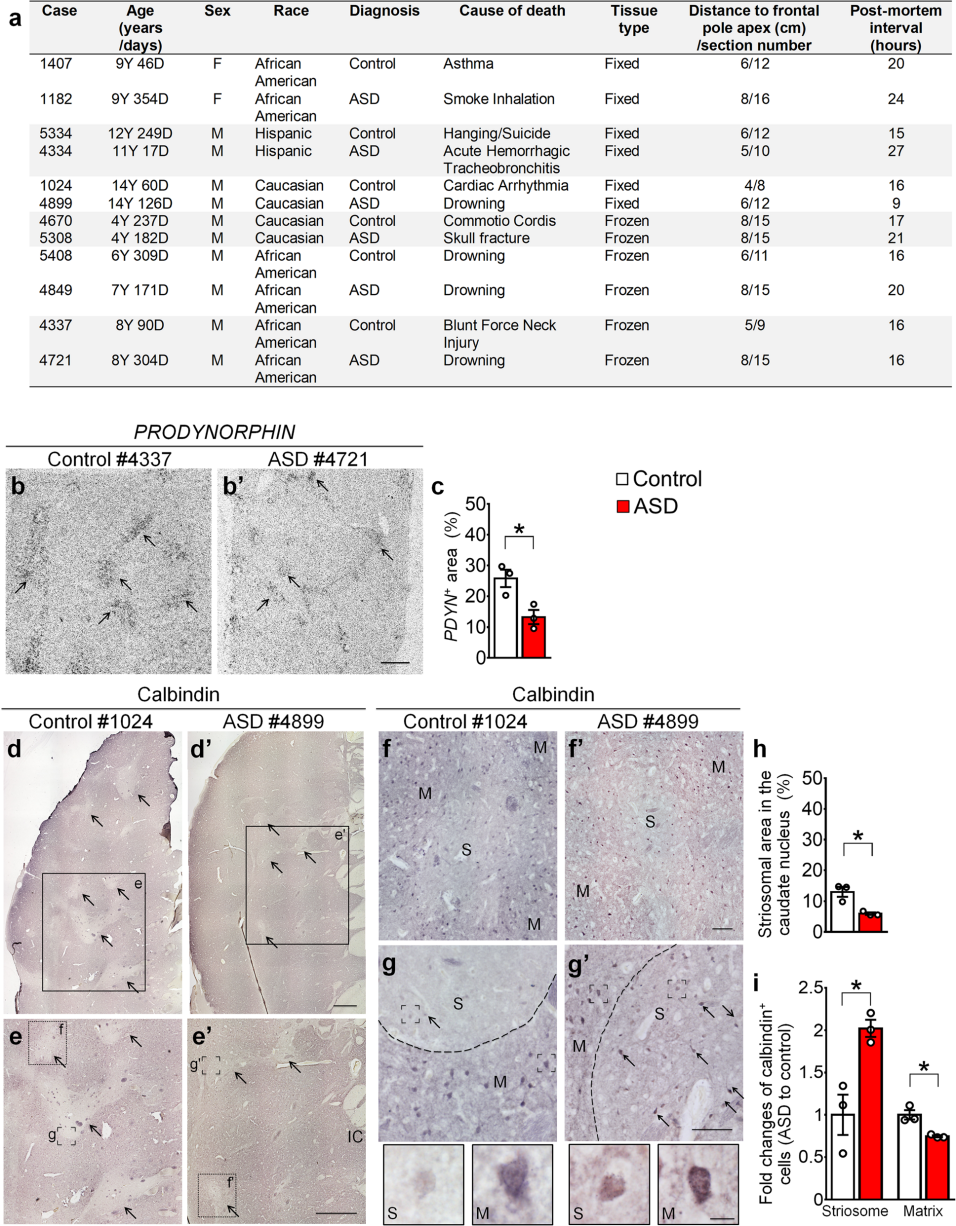
Aberrant striatal compartments in the caudate nucleus of human ASD brains. **a** Information on human subjects. The brain section numbers indicate the locations of the sampled sections relative to the frontal pole apex (See Additional file 1). **b-c** In situ hybridization shows that *PDYN*-positive striosomes (arrows) are decreased in the caudate nucleus of ASD brains (**b’**, **c**) compared to the control brains (**b**, **c**). **d-e’** The pattern of calbindin-poor striosomes (arrows) is less distinct in ASD caudate nucleus (**d’**, **e’**) than that in controls (**d**, **e**). The boxed regions in **d**, **d**’ are shown at high magnification in **e** and **e’**, respectively. **f, f’** The boxed regions in **e**, **e’** are shown at high magnification in **f**, **f’** to illustrate calbindin-poor striosomes in the control (**f**) and ASD (**f’**) brains. **g, g’** The striosomes-matrix boundaries (bracketed regions in **e**, **e’**) are shown at high magnification in **g**, **g’**. The arrows indicate calbindin-positive cells in striosomes. **h** Quantification indicates that the area of calbindin-poor striosomes is decreased, but the area of calbindin-rich matrix is increased in ASD caudate nucleus. **i** Quantification indicates that calbindin-positive cells are increased in ASD striosomes, but calbindin-positive cells are decreased in the ASD matrix compartment. *n* = 3/group. IC: internal capsule; M, matrix; S: striosome. *P < 0.05. Error bars represent s.e.m. Student’s *t*-test are used in **c**, *t*_(4)_ = 3.453 and **i**, *t*_(4)_ = − 3.962 in striosome. Mann-Whitney *U* test is used in **h**, *U* < 0.0000001 and **i**, *U* < 0.0000001 in the matrix. Scale bars: 1 mm (**b**-**b’**, **d**-**d’**, **e**-**e’**), 100 μm (**f**-**f’, g-g’**), 10 μm (high magnification panels below **f**, **f’**)

The NICHD Brain and Tissue Bank for Developmental Disorders kindly provided human postmortem brain tissue blocks of the caudate nucleus from ASD patients (*n* = 6) and control subjects (*n* = 6). Six pairs of ASD and control brains were matched for gender and race, with the ages raining from 4 to 14 years (Fig. 1a). Materials and Methods are described in Additional file 1.

We performed in situ hybridization with ^35^S-UTP-labeled probes of human *PRODYNORPHIN (PDYN)* gene, a marker of striosome/patch compartment [3]. *PDYN*-positive striosomes were present in both ASD and control caudate nucleus, but *PDYN*-positive striosomes appeared smaller in ASD caudate nucleus. Quantitative analysis indicated that the area of *PDYN*-positive striosomes was reduced by 49% in ASD brains compared to control brains (Fig. 1b, b’, c).

We further immunostained calbindin, a marker of the matrix compartment in the caudate nucleus [3, 11]. The immunoreactivity of calbindin showed typical calbindin-poor zones (striosomes) embedded in the calbindin-rich matrix of the caudate nucleus in control brains (Fig. 1d, e). Consistent with the decrease in *PDYN*-positive striosomes, the percentage of the area taken by calbindin-poor striosomes was reduced by 54% in ASD caudate nucleus compared to control brains (Fig. 1d, d’, e, e’, h). In contrast, the area of the calbindin-rich matrix compartment was increased in ASD caudate nucleus (Fig. 1d, d’, e, e’, h).

Notably, calbindin-poor striosomes were less distinct in ASD caudate nucleus than those in controls, which was likely due to an ectopic increase in calbindin-positive cells in ASD striosomes. In control brains, at most, a few weakly stained calbindin-positive cells were present in striosomes (Fig. 1f, g). In contrast, darkly stained calbindin-positive cells were increased by ~ 2 folds in ASD striosomes (Fig. 1f, f’, g, g’, i). Concurrently, we found that calbindin-positive cells in the matrix compartment were decreased in ASD caudate nucleus compared to controls (Fig. 1g, g’, h). Collectively, these results implicate pathological alterations of striatal compartmentation in young ASD brains.

The present study is the first documentation to demonstrate the abnormality of striatal compartments in the caudate nucleus of human ASD brains, which suggests abnormal striatal compartmentation as a pathological signature in ASD pathogenesis. Because of the difficulty in accessing a large number of juvenile and adolescent human ASD brains, our study was limited to a small set of ASD brains (*n* = 6 for ASD and control groups; *n* = 3 per group for in situ hybridization or immunohistochemistry). Due to the limitation of available tissue blocks from the Human Brain Tissue Bank, the anatomical levels and the post-mortem time of analyzed samples could not be exactly matched between ASD and control brains in most cases (Fig. 1a). The correlational analyses between the indexes, anatomical levels and post-mortem time showed no correlations in the control group. Nor were correlations found in the ASD group except that a correlation of calbindin-poor striosomal areas and anatomical levels was found in the ASD group (Additional file 1). Despite the correlational analyses, it remains possible that the unmatched anatomical levels or post-mortem time between the control and ASD groups may affect the results of group differences. The findings of the present study await to be confirmed with a large collection of young ASD brains.

Despite the small set of ASD brains, we found consistent pathological alterations of striosomes in ASD caudate nucleus as evidenced by the reductions in *PDYN*-expressing striosomes and calbindin-poor striosomes. Note that we cannot rule out the possibility that ASD brains had a lower number of cells in the striosomal compartment than control brains. Ectopically increased calbindin expression was also observed in striosomes of the ASD caudate nucleus. The concomitant changes of two independent striosomal markers suggest that the pathological changes are not likely due to variations in tissue preservation and other confounding factors. Notably, similar ASD-like phenotypes have been found in valproic acid-induced ASD mouse model [10], suggesting that defective striosomal compartment may constitute parts of ASD pathology.

The dysfunction of corticostriatal circuits has been implicated in the pathology of ASD [12]. In terms of corticostriatal connectivity, striosomal compartment receives the limbic-related cortical inputs, whereas the matrix compartment receives information from the sensory and motor cortex [3]. Given the specificity of input-output connectivity in striatal compartments, our current findings of aberrant striatal compartments in ASD brains are intriguing. For emotional evaluation of action-outcome, Graybiel and her co-workers have provided compelling evidence for the prelimbic cortex-striosomal circuits in control of decision making under cost-benefit conflicts [13]. Notably, striosomal compartment receives inputs from the limbic-related cortex, and then provides direct GABAergic inputs to dopamine neurons in the substantia nigra par compacta (SNc) [14]. Intriguingly, in light of the reports that the activity of the caudate nucleus of ASD patients is altered in response to reward [6], that ASD patients are impaired in predictive abilities [15], and that neuronal activity of striosomes encodes reward prediction errors [7, 8], our findings of aberrant striosomes in ASD brains suggest that dysfunctional cortico-striosomes-SNc circuits may contribute to the improper evaluation of action-outcome by ASD patients in a context of social interaction. For social communication, some ASD patients suffer from impairments in speech and language development [1]. Corticostriatal circuits are known as important for speech production [12]. Our previous study has shown concomitant deficits in isolation induced-ultrasonic vocalization, a primitive form of vocal communication, and synaptogenesis of cortico-striosomal pathways in valproic acid-induced ASD model [10], suggesting that abnormal development of cortico-striosomal circuits may be parts of ASD etiology related to impaired speech production.

Based on these different lines of observations, we postulate that neuropathological changes in striosomal circuits may contribute to the psychomotor-limbic aspect of impaired social communication and repetitive behavior in ASD pathogenesis. Future studies are required to test this hypothesis.

## Supplementary information

**Additional file 1.**

## Data Availability

Images of the caudate nucleus and raw quantitative data are included in Additional file 1.

## Abbreviations

ASD: Autism spectrum disorder
*PDYN*: *PRODYNORPHIN*
SNc: Substantia nigra pars compacta
