# Supplementary material for: Pathological alterations in striatal compartments in the human brain of autism spectrum disorder
Source: Mol Brain. 2020 May 27;13:83. doi: 10.1186/s13041-020-00624-2 (PMC7254729; doi:10.1186/s13041-020-00624-2)
Supplement: Supplementary file 1 — Additional file 1. [file 13041_2020_624_MOESM1_ESM.docx]

**Additional file 1**

**Pathological Alterations in Striatal Compartments in the Human Brains of Autism Spectrum Disorder**

**Authors:** Hsiao-Ying Kuo^1^, Fu-Chin Liu^1,2^

**Affiliation:**

^1^Institute of Neuroscience

National Yang-Ming University

Taipei 11221

Taiwan

^2^Address correspondence to:

Fu-Chin Liu, Ph.D.

Institute of Neuroscience

National Yang-Ming University

155, Sec. 2, Li-Nong Street

Taipei 11221

Taiwan

886-2-2826-7216 (phone)

886-2-2820-0259 (fax)

e-mail: fuchin@ym.edu.tw

**This file includes:**

**Materials and Methods**

**Additional Figure 1**

**Results of correlational analyses**

**Raw Quantitative Data**

**MATERIALS AND METHODS**

**Human brain tissues**

Twelve human postmortem caudate tissues of ASD patients (n = 6) and control subjects (n = 6) were kindly provided by the NICHD Brain and Tissue Bank for Developmental Disorders at the University of Maryland, Baltimore. Six pairs of brain tissue blocks were matched for gender, race, and comparable age (Fig. 1a). As indicated on the website (<https://www.medschool.umaryland.edu/btbankold/Brain-Protocol-Methods/Brain-Sectioning---Protocol-Method-2/>), the cerebrums were sectioned coronally at ~1 cm intervals beginning from the frontal pole apex and proceeding caudally. Frozen sections from the left hemisphere and fixed sections from the right hemisphere were labeled by odd numbers and even numbers, respectively. Of the six pairs that were analyzed in the present study, three pairs were fixed tissues, while the other three pairs were frozen tissues (Fig. 1a). The fixed tissue blocks were cryoprotected with 30% sucrose in 0.1 M phosphate-buffered saline (PBS) for 4-5 days, sectioned into 30 μm for immunohistochemistry. The frozen tissue blocks were fixed with 4% paraformaldehyde (PFA) in 0.1 M PBS overnight, cryoprotected with 30% sucrose in 0.1 M PBS for 4-5 days before cryostat sectioning. The cryostat sections were stored at -20°C before being processed for *in situ* hybridization and immunohistochemistry. The brain tissues of control and ASD brains were processed in parallel for *in situ* hybridization or immunohistochemistry.

***In situ* hybridization**

*In situ* hybridization was performed with ^35^S-UTP-labeled probes of human *PRODYNORPHIN (PDYN),* as previously described (1). The **brain sections were mounted to slides and post-fixed with 10% formaldehyde in potassium** phosphate-buffered saline **(KPBS) for 30 min at room temperature (RT). The brain sections were then treated with 0.2% proteinase K in 0.1 M Tris (pH 8.0) and 0.05 M EDTA (pH 8.0) for 5 min at 37**°C**, and washed by diethylpyrocarbonate (DEPC)-treated H_2_O. After acetylation by 0.25% acetic anhydride in 0.1 M triethanolamine, the sections were washed by 2X saline-sodium citrate buffer (SSC). They were then dehydrated by successive 3-min rinses in** 50%, 70%, 95% ethanol, followed by three rinses in 100% ethanol. ^35^S uridine 5’-(α-thio) triphosphate-labeled anti-sense RNA probes were prepared by *in vitro* transcription of the linearized human *PRODYNORPHIN* plasmid (kindly provided by Dr. Yasmin Hurd at the Mount Sinai Hospital, USA) (2, 3) with T7 RNA polymerase (Promega, USA) and 0.125 mCi ^35^S-UTP (PerkinElmer, USA). The hybridization solution contained ^35^S-UTP-labeled probes (10^7^ counts/min) were heated at 65°C for 5 min and cooled down on ice prior to hybridization. After hybridization for 16 hr at 58°C, the sections were washed with 4X SSC for 4 X 7 min and treated with 0.3% RNase A in 0.5 M NaCl, 0.01 M Tris (pH 8.0) and 1 mM EDTA (pH 8.0) for 30 min at 37°C. The sections were then washed with 2X SSC, 1X SSC, and 0.5X SSC for 5 min each at RT, 0.1X SSC for 30 min at 50°C, and 0.1X SSC for 5 min at RT. The SSC washing buffers above contained 1 mM dithiothreitol dissolved in 0.01 M sodium acetate. After dehydration by ethanol, the sections were apposed to X-ray film for the detection of ^35^S-labeled probe signals.

**Immunohistochemistry**

Brain sections were pretreated with 0.05% citraconic anhydrate for 45 min at 95°C prior to immunostaining (4). Sections were washed with 3 x 5 min with 0.1 M PBS and then treated with 0.2% Triton X-100 in 0.1 M PBS (PBST) for 10 min and 3% H_2_O_2_ and 10% methanol in 0.1 M PBST for 5 min. After incubation with 3% normal goat serum in 0.1 M PBS for 1 hr, sections were incubated in mouse anti-calbindin antibody (1:500, Swant, #300) for three overnight at 4°C. After washing 3 x 5 min with 0.1 M PBS, sections were incubated with biotinylated goat anti-mouse antibody (Vector Laboratories) for 1 hr. After 3 x 5 min rinses with 0.1 M PBS, sections were treated with avidin-biotin-peroxidase complex (PK-6100, Vector Laboratories) for 1 hr. Sections were developed in 0.1 M PB containing 0.02% diaminobenzidine, 0.08% nickel ammonium sulfate and 0.003% final concentration of H_2_O_2_.

**Statistical analysis**

The Shapiro-Wilk test was applied to assay the normal distribution of data. For the data that met the assumption of normality, the data were analyzed by the Levene’s test for equality of variances, followed by the Student's two-sided *t*-test. For the data that did not meet normality, the data were processed with the Mann-Whitney *U* test. Raw quantitative data are shown in Additional Figure 1. *PDYN*-positive or calbindin-poor striosomal areas were divided by the total area of the caudate nucleus (Fig. 1c, h). The numbers of calbindin-positive cells in striosomal or matrix compartment were counted, and they were then divided by the area of striosomal or matrix compartment to derive the density of calbindin-positive cells. The calbindin-positive cells density in each compartment was divided by the calbindin-positive cells density in the whole caudate nucleus. These ratio values of the ASD group were then expressed as fold changes relative to the control group (Fig. 1i). Data were plotted and presented as mean ± s.e.m. in the bar graphs. Correlational analyses between the indexes, anatomical levels, and post-mortem time were performed with Pearson correlation. For the data that did not meet normality, the data were processed with the Spearman rank correlation. Quantitative measurements were performed using ImageJ software (http://imagej.nih.gov/ij/). Statistical analyses were performed using SPSS Statistics 21 software (IBM, Armonk, New York, United States).

**REFERENCES**

1. Liao WL, Tsai HC, Wang HF, Chang J, Lu KM, Wu HL, et al. Modular patterning of structure and function of the striatum by retinoid receptor signaling. Proc Natl Acad Sci U S A. 2008;105(18):6765-70.

2. Hurd YL. In situ hybridization with isotopic riboprobes for detection of striatal neuropeptide mRNA expression after dopamine stimulant administration. Methods Mol Med. 2003;79:119-35.

3. Hurd YL. Differential messenger RNA expression of prodynorphin and proenkephalin in the human brain. Neuroscience. 1996;72(3):767-83.

4. Alelu-Paz R, Iturrieta-Zuazo I, Byne W, Haroutunian V, Garcia-Villanueva M, Rabano A, et al. A new antigen retrieval technique for human brain tissue. PLoS One. 2008;3(10):e3378.


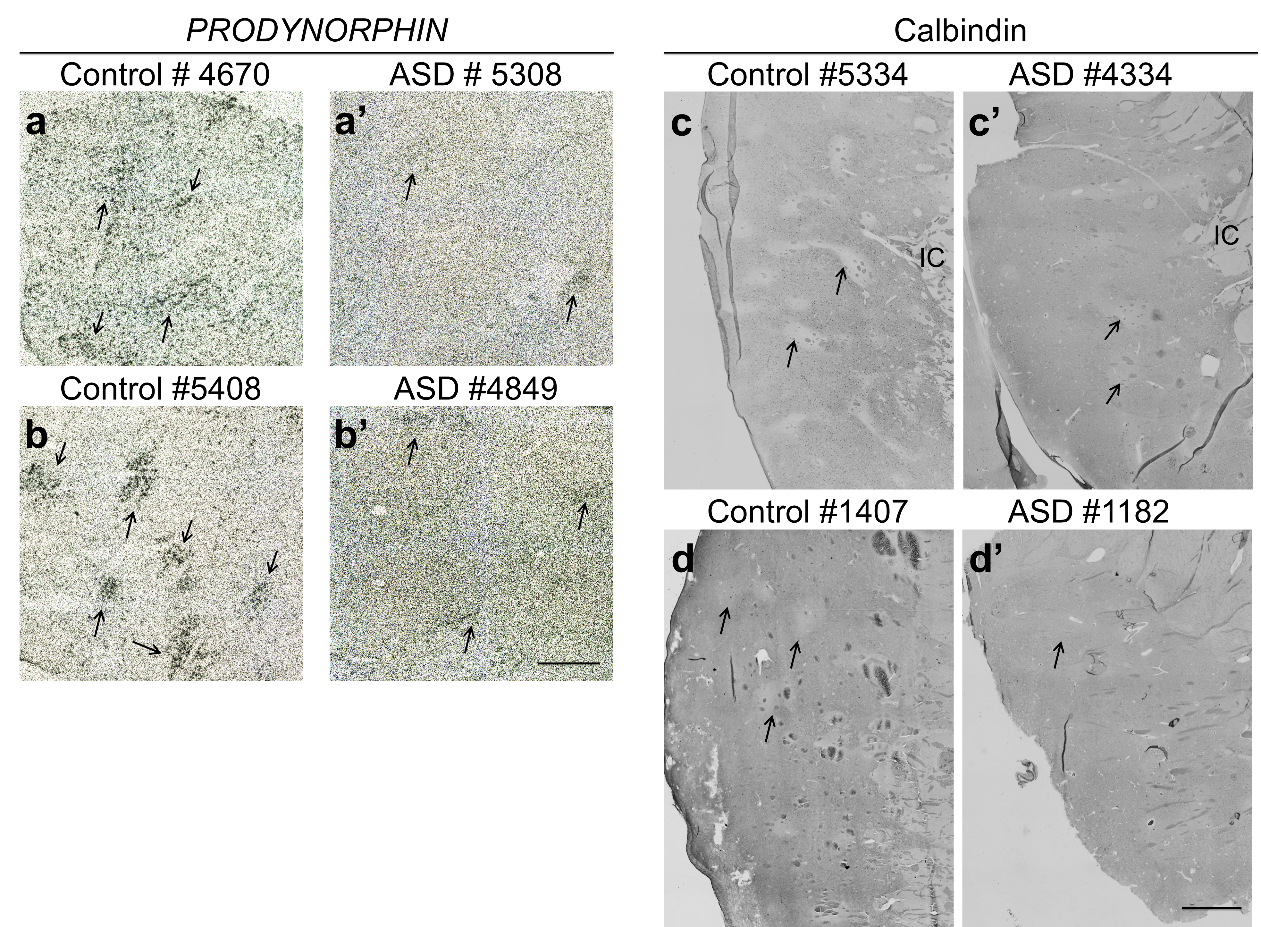


**Additional Figure 1.** **Photomicrographs of *PDYN*-positive and calbindin-poor striosomes in the other two pairs of ASD and control brains. (a-b’)** *PDYN*-positive striosomes (arrows, **a’**, **b’**) are decreased in ASD caudate nucleus (ASD #5308, #4849) compared to control caudate nucleus (**a, b**: Control #4670, #5408). **(c-d’)** Calbindin-poor striosomes (arrows) are less distinct in ASD brains **(c’, d’)** than control brains **(c, d)**. PDYN: prodynorphin. Scale bars: 1 mm (**a-a’**, **b**-**b’**), 2 mm (**c-c’**, **d**-**d’**).

**Results of correlational analyses**

**Correlation between each parameter and the distance to the frontal pole apex**

*The correlation cannot be computed because the frozen tissue blocks of the ASD caudate nucleus are at the same anatomical level.

**Correlation between each parameter and the post-morterm intervals**

**Raw Quantitative Data**

**Raw data of the bar graphs in Figure 1**

**Cell counts of calbindin-positive cells in striosomal and matrix compartments and the whole caudate nucleus**

**Areas of striosomal and matrix compartments and the entire caudate nucleus**

**Densities of calbindin-positive cells in striosomal, matrix compartments and the whole caudate nucleus**

**Ratios of calbindin-positive cell densities in striosomal and matrix compartments relative to that in the entire caudate nucleus**
